# On Being the Right Size: The Impact of Population Size and Stochastic Effects on the Evolution of Drug Resistance in Hospitals and the Community

**DOI:** 10.1371/journal.ppat.1001334

**Published:** 2011-04-14

**Authors:** Roger D. Kouyos, Pia Abel zur Wiesch, Sebastian Bonhoeffer

**Affiliations:** 1 Institute of Integrative Biology, ETH Zurich, Zurich, Switzerland; 2 Department of Ecology and Evolutionary Biology, Princeton University, Princeton, New Jersey, United States of America; Emory University, United States of America

## Abstract

The evolution of drug resistant bacteria is a severe public health problem, both in hospitals and in the community. Currently, some countries aim at concentrating highly specialized services in large hospitals in order to improve patient outcomes. Emergent resistant strains often originate in health care facilities, but it is unknown to what extent hospital size affects resistance evolution and the resulting spillover of hospital-associated pathogens to the community. We used two published datasets from the US and Ireland to investigate the effects of hospital size and controlled for several confounders such as antimicrobial usage, sampling frequency, mortality, disinfection and length of stay. The proportion of patients acquiring both sensitive and resistant infections in a hospital strongly correlated with hospital size. Moreover, we observe the same pattern for both the percentage of resistant infections and the increase of hospital-acquired infections over time. One interpretation of this pattern is that chance effects in small hospitals impede the spread of drug-resistance. To investigate to what extent the size distribution of hospitals can directly affect the prevalence of antibiotic resistance, we use a stochastic epidemiological model describing the spread of drug resistance in a hospital setting as well as the interaction between one or several hospitals and the community. We show that the level of drug resistance typically increases with population size: In small hospitals chance effects cause large fluctuations in pathogen population size or even extinctions, both of which impede the acquisition and spread of drug resistance. Finally, we show that indirect transmission via environmental reservoirs can reduce the effect of hospital size because the slow turnover in the environment can prevent extinction of resistant strains. This implies that reducing environmental transmission is especially important in small hospitals, because such a reduction not only reduces overall transmission but might also facilitate the extinction of resistant strains. Overall, our study shows that the distribution of hospital sizes is a crucial factor for the spread of drug resistance.

## Author Summary

The increasing spread of bacteria, which are resistant to antibiotics, is a serious threat to clinical care. Currently, several countries aim at concentrating highly specialized services in large hospitals in order to improve patient outcomes. However, empirical studies have shown that resistance levels correlate with hospital size. To illustrate this correlation, we analyze two published datasets from the US and Ireland and controlled for antimicrobial usage, disinfection and length of stay. The proportion of patients acquiring both sensitive and resistant infections in hospitals strongly correlated with hospital size. Moreover, we observe the same pattern for both the percentage of resistant infections and the temporal increase of hospital-acquired infections. To investigate to what extent hospital size can directly affect the prevalence of antibiotic resistance, we use mathematical models describing the epidemic spread of resistance in hospitals and the community. We find that small hospitals typically lead to considerably lower resistance levels than large hospitals. However, this beneficial effect of small hospital size may be reduced if bacteria are transmitted indirectly via the environment. Therefore, reducing environmental transmission might be particularly important in small hospitals. Overall, our findings suggest that the short-term benefits of larger hospitals may come at the price of increasing resistance in the long term.

## Introduction

The last decades have shown that the introduction of an antibiotic agent is almost inevitably followed by the spread of resistance mutations that jeopardize the beneficial effect of this agent [1], [2]. Because of this process of bacterial adaptation to antibiotics, maintaining the benefits of antibiotic therapy requires a steady development of new drugs or drug classes. Population biological models may contribute to slowing down the required pace of this “drug treadmill” by identifying the factors that determine the adaptability of bacterial populations to antimicrobial treatment [3].

The epidemic spread of antibiotic resistance can be strongly affected by the structure of the human host population[4]. One of the most important instances of such population structure is the interaction between the hospital and the community[4]. These two settings differ with respect to several parameters that are crucial for the spread of antibiotic resistance. While the hospital environment is characterized by small population sizes, high transmission rates, fast turn over and frequent use of antibiotics, the community exhibits comparatively large population sizes, small transmission rates, slow turn-over rates and infrequent use of antibiotics. Hospitals are often the source of emergent resistant strains [5], but this spread is not unidirectional, as illustrated by outbreaks of community-acquired MRSA in health care facilities [6]. Since the spread of resistance mutations [7] increases with antibiotic usage [8], the difference in treatment frequencies may explain why hospitals mostly act as source for resistance mutations and the community acts as a sink.

The size distribution of hospitals is an important determinant for the population structure generated through the hospital-community interaction. In small hospitals, bacterial population sizes and frequencies are subject to strong stochastic effects and populations may frequently become extinct. It has been empirically shown that resistance levels tend to be lower in small hospitals [9], [10], [11], [12], [13]. In principle, this can be due to two reasons: On the one hand, small hospitals might be associated with different types of patients and treatments (i.e. lower antibiotic usage [14]), which select for less resistance. On the other hand, small hospital size by itself might hinder bacterial adaptation and thereby reduce resistance levels. The fact that the analysis in [10] controlled for patient characteristics, suggests that, at least in that case, the impact of hospital size was due to the second “intrinsic” mechanism. In this study we use a simple population biological model to analyze the intrinsic effects of hospital size, i.e. we assess to what extent the stochastic effects resulting from small hospital populations may help in alleviating the burden of antibiotic resistance.

## Methods

**Figure 1:**
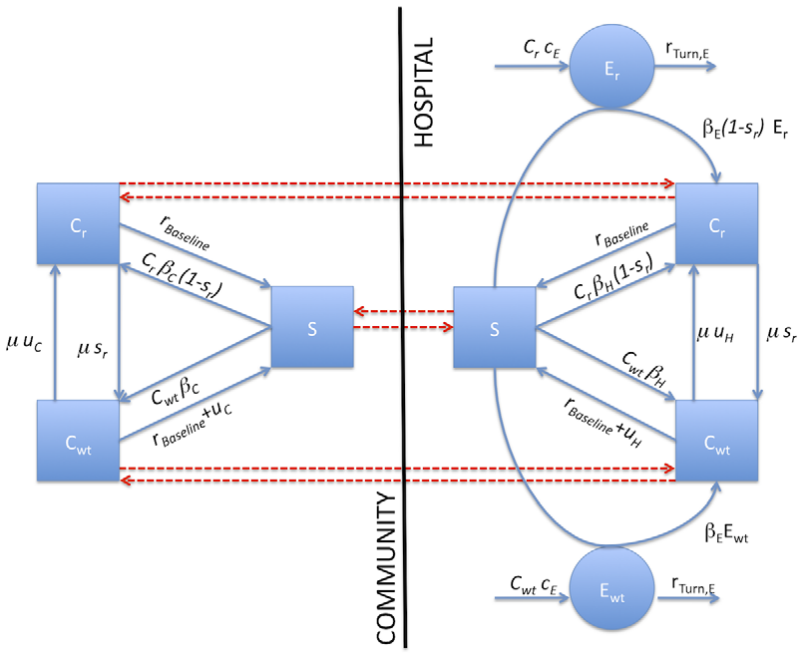
Flow chart of compartmental model in the simplest case (only one hospital and one community).

**Figure 2:**
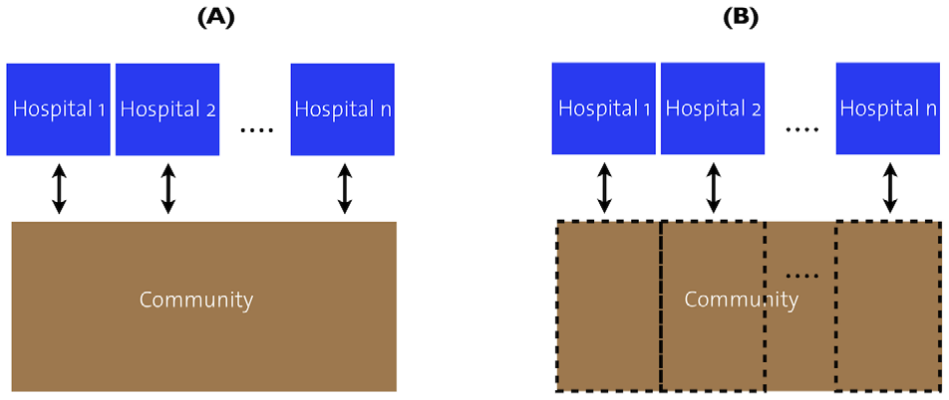
Illustration of the panmictic and subdivided population structures. Illustration of the models with panmictic (A) and subdivided (B) community.

The model presented here extends the basic models for the spread of antibiotic resistance [15], [16] to a setting in which several hospitals interact with the community. The flowchart in figure 1 shows the model in the simplest case (one hospital and one community). More complicated structures of communities and hospitals are illustrated in figure 2. The code was implemented in the programming language C, the statistical programming package R was used for the graphical representation of the results. The parameters used and the corresponding references are listed in table 1. As hospitals are often characterized by small population sizes, we describe populations stochastically. Specifically, we use the tau-leap approximation [17] of the Gillespie algorithm [18] to implement stochasticity. The host-population is compartmentalized according to location (community, hospital 1, hospital 2, …, hospital n) and colonization status (uncolonized; colonized with sensitive strain; colonized with resistant strain). The model includes the following processes:

**Table 1:** Default model parameters of the model.

Parameter Name	Explanation	Default Value
T	Observation period over which results are averaged	30*365 d
E_H_	Number of beds of one hospital	10–500
E_C_	Size of community	300*Total Hospital Size#
β_H_	transmission rate in hospital	0.4 d^−1^/Number of Beds
β_C_	transmission rate in community	0.005 d^−1^/Size of community
s	cost of drug resistance	10% –30% [28], [29], [30]
μ	Mutation rate	10^−3^ d^−1^
r_Turn_	Turn-over rate of patients in hospital (i.e. discharge rate)	1/7 d^−1^ ##
r_Baseline_	Baseline clearance rate	1/300 d^−1^ [32]
u_H_	Frequency of antibiotic use in hospital	0.005–0.5 [33]
u_C_	Frequency of antibiotic use in the community	10^−4^–0.02 [34]
*Extension with environmental transmission in the hospital*		
c_E_	Colonization rate of environment	1/10
r_Turn,E_	Turn-over rate of environment	1/30 [22]
β_E_	Transmission rate from environment	β_H*_ r_Turn,E_/c_E_
*Extension with inter-ward transfer*		
w	Inter-ward transfer rate	0.01–0.1/patient day [23]

# Global average number of hospital beds per inhabitant, as retrieved from the WHO database http://apps.who.int/ghodata/.

## The average length of stay is 8 days in Switzerland (http://www.obsandaten.ch/indikatoren/5_4_1/2005/d/541.pdf, data from 2005) and 5 days in the US (http://www.cdc.gov/mmwr/preview/mmwrhtml/mm5427a6.htm).

- *Admission & Discharge*: Hospitals are characterized by the turnover-rate of patients (*r_Turn_*), the expected patient population size for which the hospital has been conceived (number of beds)(*S_H_*) and the actual number of patients at a given time point (*N_H_*). Admission and discharge events occur at rates *S_H_ r_Turn_* and *N_H_ r_Turn_,* respectively, so that *S_H_* corresponds to the average number of patients. At an admission event a randomly chosen patient from the community enters the hospital and at a discharge event a randomly chosen patient leaves the hospital and reenters the community. We assume that the colonization status does not affect the admission or discharge rates. This is a reasonable approximation for many endogenous infections, where organisms that are usually part of the commensal microflora enter previously sterile body compartments.
- *Colonization:* Uncolonized patients in the hospital and the community become colonized with strain *i* (here and below *i* can be either *i  =  s* for the sensitive or *i  =  r* for the resistant strain) with a rate *N_H,i_ β_H_ (1-s_i_)* and *N_C,i_ β_C_ (*1*- s_i_),* respectively. Here *N_H,I_* (*N_C,_*_i_) denotes the number of patients in the hospital (community) infected with strain *i, β_H_ (β_C_)* denotes the transmission rate in the hospital (community), and *s_i_* the fitness costs of the mutations carried by strain *i* (i.e. *s_i_* is equal to 0 for the sensitive strain and equal to the cost of resistance for the resistant strain).
- *Clearance:* In the hospital, patients colonized with strain *i* are cleared with a rate *r_Baseline_*+*u_H_* if strain *i* is susceptible and with a rate *r_Baseline_* is strain *i* is resistant. Here, *r_Baseline_* denotes the baseline clearance and *u_H_* the usage frequency of the antibiotic in the hospital. For the community, clearance rates are obtained by substituting the usage frequency in the hospital by the usage frequency in the community (*u_C_*).
- *Mutation*: A mutation from a strain *i* to a strain *j* becomes fixed in a patient if it increases replicative fitness, i.e. if it either confers resistance in a treated patient or a growth advantage in an untreated patient. In the first case the mutation occurs with a rate *N_H,j_ μ u_H_* and in the second case it occurs with a rate *N_H,j_ μ (s_i_ -s_j_)* for the hospital and analogously for the community.

Each simulation run consists of a burn-in period followed by a treatment period. In the burn-in period, the model is simulated for 30 years in absence of treatment (in order to reach the treatment-free equilibrium, which typically consists in the absence of resistant strains). In the treatment period, antibiotic usage frequencies (*u_H,_ u_C_*) increase linearly in the first five years until they reach their final value, which is kept constant for the remaining 25 years of the treatment period. In the following, values given for antibiotic usage frequencies always refer to this final value.

### Environmental transmission

Environmental transmission in the hospital can be added to the above model via two additional, deterministic compartments corresponding to the sensitive and resistant strain: the density of bacteria of strain *i* in the environment, denoted E_i_. Bacteria of strain *i* colonize the environment with a rate *N_H,i_ c_E_* and are cleared from the environment at a constant rate r_Turn,E_. Bacteria of strain *i* from the environment can in turn infect susceptible patients with a force of infection *β_E_ E_i_*_._. Because the total number of bacteria in the environment is presumably very large and the dynamics of the environmental compartment are not directly affected by the fluctuations in the patient population, we assume that this compartment can be adequately described deterministically.

## Results

In this study we consider the effect of hospital size distribution on the epidemic spread of antibiotic resistance. Specifically, we assess whether from the point of view of resistance prevention/minimization, it is preferable to have a small number of large hospitals or a large number of small hospitals. The focus of this study is the theoretical assessment of resistance spread in hospitals of different sizes. We start however with an analysis of two published surveillance datasets from the US and Ireland to illustrate the correlation between hospital size and acquisition rates of both sensitive and resistant strains.

### Analysis of surveillance data

**Figure 3:**
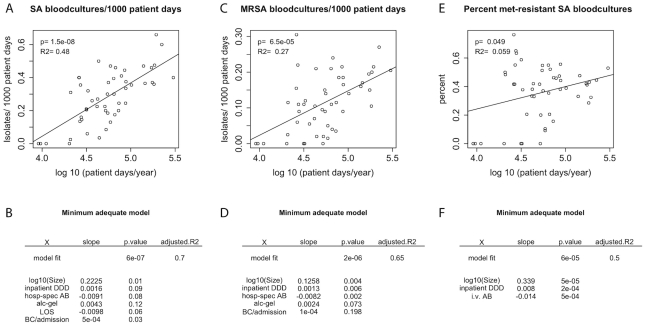
Correlations between hospital size and incidence rates or resistance in Ireland 2006–2007. *Size =  Patient days/year; inpatient DDD =  Mean defined daily doses given to inpatients per 100 patient days; hosp-spec AB =  Mean defined daily doses of hospital specific antibiotics/100 patient days; in. AB =  Mean defined daily doses of injectable antibiotics/100 patient days; alc-gel =  Mean consumption of alcohol-based hand gels in L/1000 patient days; BC/admission =  Mean number of bloodcultures per 1000 admissions.* A) Mean total incidence rate of blood cultures positive for S. aureus as a function of hospital size, the line represents the univariate linear model of incidence rate against hospital size B) Minimum adequate model explaining the total incidence rate C) Mean incidence rate of blood cultures positive for methicillin-resistant S. aureus (MRSA) as a function of hospital size, the line represents the univariate linear model of incidence rate against hospital size D) Minimum adequate model explaining the incidence rate of MRSA E) Mean percentage of MRSA among all S. aureus bloodstream isolates F) Minimum adequate model explaining the percentage of methicillin-resistant infections as a function of hospital size, the line represents the univariate linear model of percent resistant isolates against hospital size.

We used an Irish surveillance dataset published by the Health Service Executive Ireland [19] to investigate to what extent the incidence of infections, especially by resistant strains, correlates with hospital size. This dataset contains information from 53 hospitals about both the total number of new infections with S. aureus as well as infections with MRSA (all positive blood-cultures were recorded). Additionally reported quantities were: length of stay, total inpatient antibiotic usage, injectable inpatient antibiotic usage, the usage of hospital-specific antibiotics, consumption of alcohol hand-gel and the frequency of blood cultures per admission. We found a significant correlation between the number of patient days/year (which is a proxy for hospital size), and the rate of both total (figure 3A) and resistant (figure 3C) S. aureus acquisitions, as well as the percentage of methicillin-resistant isolates among all S. aureus positive blood cultures (see figure 3E). This correlation remained significant even when controlling for all the above-mentioned variables (figure 3B,D,F). In the minimum adequate model chosen on the basis of the Akaike information criterion (as implemented in the function *step(lm())* in R), hospital size was the parameter which overall had the most significant impact. Unsurprisingly, the amount of overall antibiotic usage also strongly correlated with both absolute and relative resistance levels. Nevertheless, the model fits (Figure 3) suggest that the impact of hospital size on resistance level is at least of similar magnitude than the impact of antibiotics consumption.

**Figure 4:**
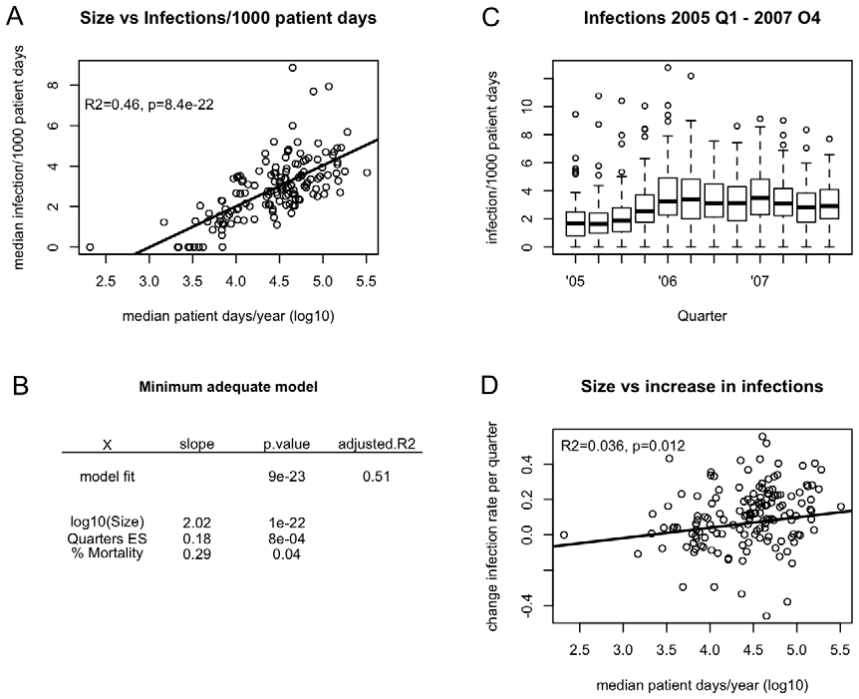
Correlations between hospital size and incidence rates and their increase in Pennsylvania 2005–2007. A) Median quarterly incidence rate of nosocomial (>48h after admission) infections as a function of hospital size, the line represents the univariate linear model of incidence rate against hospital size B) Minimum adequate model explaining the incidence rate of nosocomial infections C) Quarterly incidence rates of noscomial infections in Pennsylvania 2005–2007 D) Change in incidence rates from 2005–2007. Here, a linear model was fitted to the quarterly incidence rates for each hospital. For each hospital, the slope of this regression (controlled for the introduction of electronic surveillance) was plotted against the number of patient days per year. In order to determine the correlation between the change in incidence rates and hospital size, the slopes were set to zero if there was no significant trend during the observed period.

If smaller hospitals had lower resistance levels due to extinction events, the same should generally be true for the total incidence of hospital-acquired infections (although to a lesser degree, since the number of total infections is always higher than the one of resistant infections). To test our findings obtained with the Irish dataset, we used surveillance data from the Pennsylvania Health Care Cost Containment Council (PHC4) [20] to investigate in how far infection rates are proportional to hospital sizes. In total, 149 Hospitals reported nosocomial infection rates (i.e. infections that became symptomatic >48h after admission) quarterly from 2005 to 2007. Furthermore, mortality rates, mean length of stay and infection rates by disease type (e.g. bloodstream infection or pneumonia) were reported once per year. The median infection rate throughout these 12 quarters strongly correlated with hospital size (see figure 4A). This correlation remained very strongly significant even when accounting for potential confounders such as length of stay, mortality or the number of quarters in which electronic surveillance was used (see figure 4B). In the minimum adequate model, the usage of electronic surveillance also had a significant influence on infection rates (see figure 3B). This is presumably because a higher fraction of infections is reported with electronic surveillance. However, even in this analysis the most significant effect (i.e. smallest p-value) on resistance levels was due to hospital size. We observed that infection rates and hospital size were significantly correlated in any given year for the total infection rate as well as for the infection rates of most types of infections (see table 2). Apart from the average level of infection rates, one would also expect that the temporal increase of infection rates would be smaller with frequent extinction events. During the years 2005–2007, there was a slight overall increase in infection rates (see figure 4C). For each hospital, we fitted the total rate of acquiring infection with a linear model with time and presence of electronic surveillance as explanatory variables. If the change over the three years was not significantly correlated to time, we set this change to zero. Also the change in infection rates was significantly correlated to the logarithm of the hospital size (see figure 4D).

**Table 2:** Correlation between hospital size and type of infection for each given year from 2005–2007.

	2005			2006			2007		
	R^2^	slope	p-value	R^2^	slope	p-value	R^2^	slope	p-value
***Total Infections***	0.3	7.1	1×10^−14^ (***)	0.4	11.4	4×10^−20^ (***)	0.41	9.7	8×10^−21^ (***)
**Urinary Tract**	0.22	4.0	7×10^−11^ (***)	0.34	5.4	2×10^−16^ (***)	0.28	4.4	2×10^−13^ (***)
*Urinary Tract (device-associated)*	*no data*			*0.29*	*4.0*	*6*×*10^−14^ (* *** *)*	*0.32*	*3.4*	*2*×*10^−15^ (* *** *)*
*Urinary Tract (nondevice-associated)*	*no data*			*0.12*	*1.4*	*4*×*10^−06^ (* *** *)*	*0.05*	*1.0*	*0.0032 (* ** *)*
**Pneumonia**	0.03	0.4	0.0088 (**)	0.02	0.5	0.0579	0.08	0.8	0.0001 (***)
*Pneumonia (device-associated)*	*no data*			*0*	*−0.2*	*0.2374*	*0.05*	*0.2*	*0.0024 (* ** *)*
*Pneumonia (nondevice-associated)*	*no data*			*0.06*	*0.7*	*0.0009 (* *** *)*	*0.05*	*0.6*	*0.0029 (* ** *)*
**Bloodstream**	0.17	1.3	2×10^−08^ (***)	0.22	1.4	1×10^−10^ (***)	0.22	1.1	1×10^−10^ (***)
*Bloodstream (device-associated)*	*no data*			*0.13*	*0.9*	*7×10^−07^ (* *** *)*	*0.12*	*0.6*	*3×10^−06^ (* *** *)*
*Bloodstream (nondevice-associated)*	*no data*			*0.18*	*0.5*	*5×10^−09^ (* *** *)*	*0.18*	*0.5*	*8×10^−09^ (* *** *)*
**Surgical Site**	0	1.0	0.249	−0.01	0.3	0.6918	0	−0.4	0.5343
**Gastrointestinal**	no data			0.13	1.7	9×10^−07^ (***)	0.18	1.4	1×10^−08^ (***)
**Other Infections**	no data			0.03	0.2	0.0132 (*)	0.02	0.2	0.0524
**Multiple**	0.09	0.9	7×10^−05^ (***)	0.24	1.9	2×10^−11^ (***)	0.27	1.5	3×10^−13^ (***)

*p<0.05,

**p<0.01,

***p<0.001

### Theoretical interpretation

We mainly consider two simple settings (see Figure 2), in which hospitals of equal size are linked to a community, which is either panmictic or strongly subdivided. In the case of a subdivided community, we allow for migration between the sub-communities (migration rate between 1%/year and 20%/year). These two settings can be considered as models of the population structure in urban and rural environments respectively. In either case we assume that the number of hospital beds per inhabitant (i.e. the fraction between the total hospital size and the community size) is constant. Specifically, we consider a community of 3*10^5^ individuals with 1000 hospital beds.

**Figure 5:**
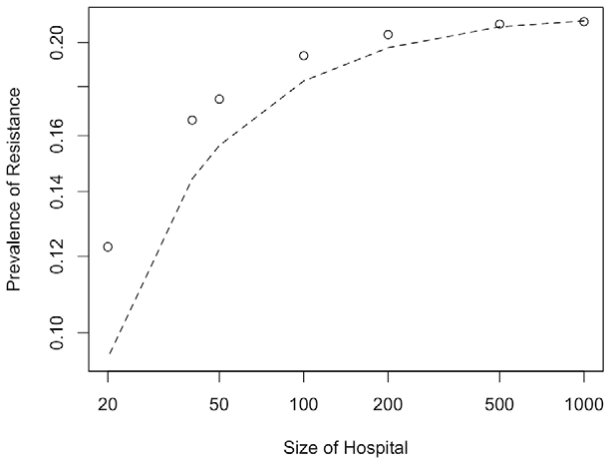
The effect of hospital size. Prevalence of resistance in the hospital for hospitals of different sizes linked to a subdivided (dashed lines) or panmictic community (dots). Antibiotic usage frequency is high in the hospital (*u_H_* =  0.1), but low in the community (*u_C_*  =  0.0001). For the subdivided community the rate of migration between sub-communities is 5%/year. Each point corresponds to the average over 1000 simulations.

We find that, if drug use is high in the hospital and low in the community, the level of resistance increases with hospital size (Figure 5). This effect is even more pronounced if the community is subdivided as well. However, substructure in the community only seems to have a minor impact compared to the population structure of hospitals (Figure 5). This makes sense intuitively as population sizes in the community are much larger and hence stochastic effects are comparatively weak. The pattern in figure 5 represents the typical situation for a nosocomial pathogen where treatment rates are high in the hospital but low in the community. In the following we generalize this pattern to a broad range of treatment frequencies. We first describe the case of a panmictic community, which as Figure 5 suggests represents the conservative scenario concerning the effect of population size, and then consider the additional effects conferred by substructures in the community.

#### Effects of hospital size in a panmictic community

**Figure 6:**
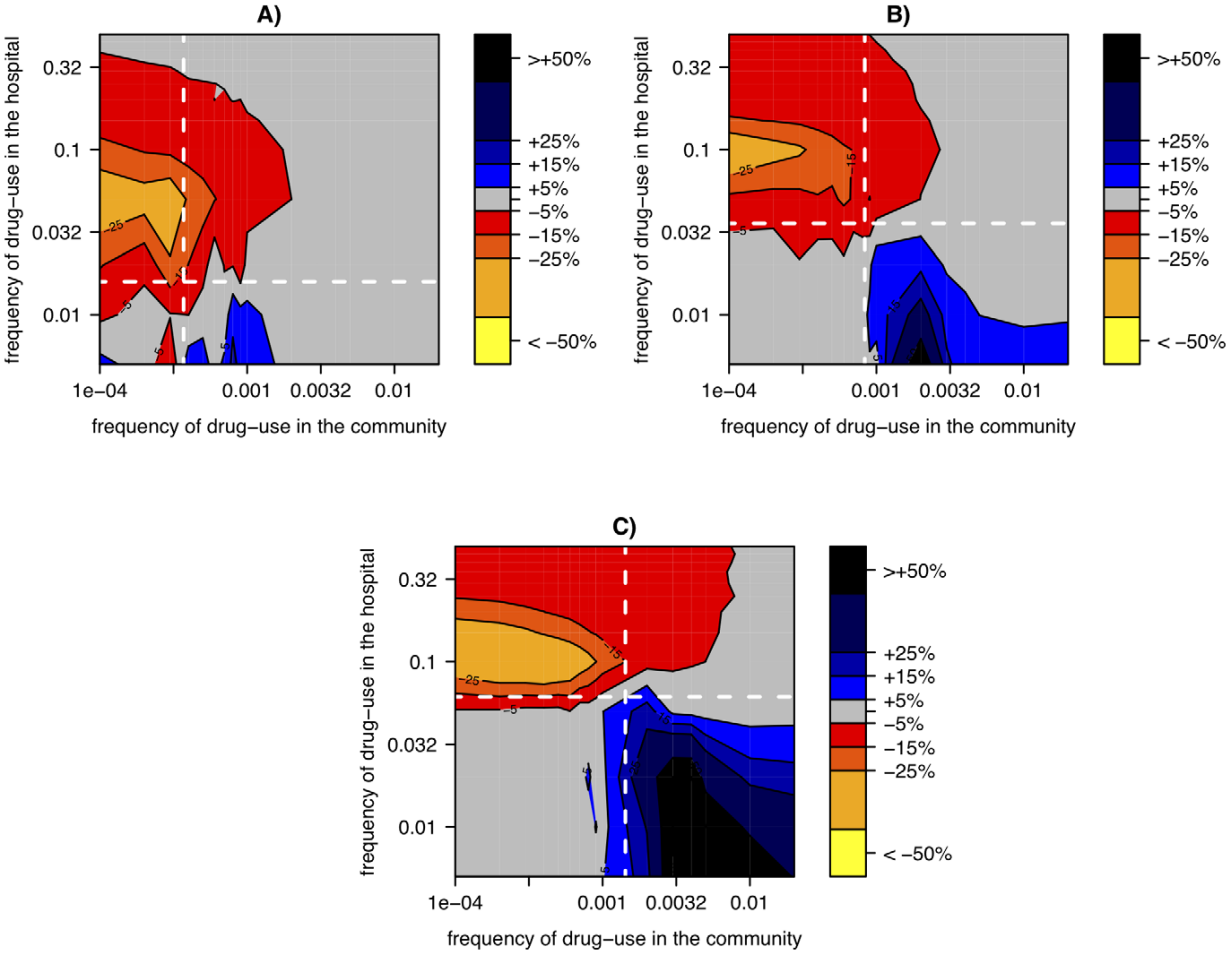
Relative change of resistance-prevalence in small vs. large hospitals linked to a panmictic community. The relative change in resistance-prevalence is measured as 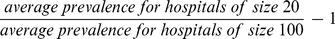 and is plotted as a function of the frequency of drug-use in the hospital and the community. Panels correspond to different costs of drug resistance: *s = 0.1 (A), s = 0.2 (B), s = 0.3 (C).* The resistance prevalence for each parameter combination has been computed as the average over 500 simulations. The white lines correspond to the threshold values when R0 of the resistant strain becomes higher than R0 of the wild-type strain in the hospital (horizontal) and the community (vertical).

When varying the usage frequencies of antibiotics, we find three basic effects (Figure 6A): 1) Reducing hospital size leads to a substantial reduction of resistance prevalence if the antibiotic is deployed frequently in the hospital but infrequently in the community. 2) This beneficial effect of small hospital size decreases in magnitude if drug-use in the hospital is too large (upper left corner in Figure 6A, B and C). 3) Contrary to the pattern in figure 5, small hospital size might lead to an increase in the level of resistance if antibiotic usage frequencies are of similar magnitude in both the hospital and the community. The effects 1–3 also occur for different fitness costs (Figure 6A–C), although the exact usage frequencies at which they occur shifts.

These effects and the parameter combinations at which they occur can be understood as follows: If antibiotic usage is low in the community and high in the hospital, selection acts strongly against antibiotic resistance in the community and in favor of antibiotic resistance in the hospital. In this case, frequent extinctions in the hospital, caused by a small population size, weaken the resistance-favoring selection in the hospital compared to the resistance-disfavoring selection in the community. This occurs because, when the resistant strain is extinct in the hospital, selection has no diversity to act on and is therefore ineffective[21]. Hence, with decreasing hospital size, resistance occurs at lower levels (Effect 1). Since the hospital acts as a source for the drug resistance mutations in the community, the prevalence of drug resistance in the community increases with increasing drug usage in the hospital. Thus, if drug usage in the hospital is too large then drug resistance becomes frequent in the community. This in turn impedes extinctions in the hospital through frequent reintroduction of resistant strains and accordingly reduces the impact of hospital size (Effect 2). Finally, Effect 3 can be understood as the inversion of Effect 1, which occurs when resistance is selected against in the hospital and selected for in the community (see below).

In this context it is important to note that, because of different turnover rates, a level of drug usage that would lead to selection against drug resistance in the hospital can lead to selection for drug resistance in the community. Selection in the hospital or the community on its own, favors resistance if the fitness of the resistant strain (R0') exceeds the fitness of the sensitive strain (R0). The ratio of these fitnesses is given by
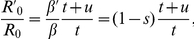
where *β* (*β’*) denotes the transmission rate of the sensitive (resistant strain), *t* the turnover of infections in the setting considered, *s* the fitness cost of drug resistance, and *u* the usage frequency of the antibiotic. It follows that resistance is selected (i.e. R0'/R0>1) if the usage frequency is above a threshold given by 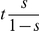. As the turnover in the hospital is much larger than the turnover in the community (here 1/7 *d* ^−1^ vs. 1/300 *d* ^−1^) the same applies to these thresholds and hence resistance becomes selected for at much lower usage frequencies in the community than in the hospital. The white lines in Figure 6 correspond to these threshold values and show that these thresholds can indeed largely explain the patterns 1 and 3.

#### Additional effects of substructures in the community

**Figure 7:**
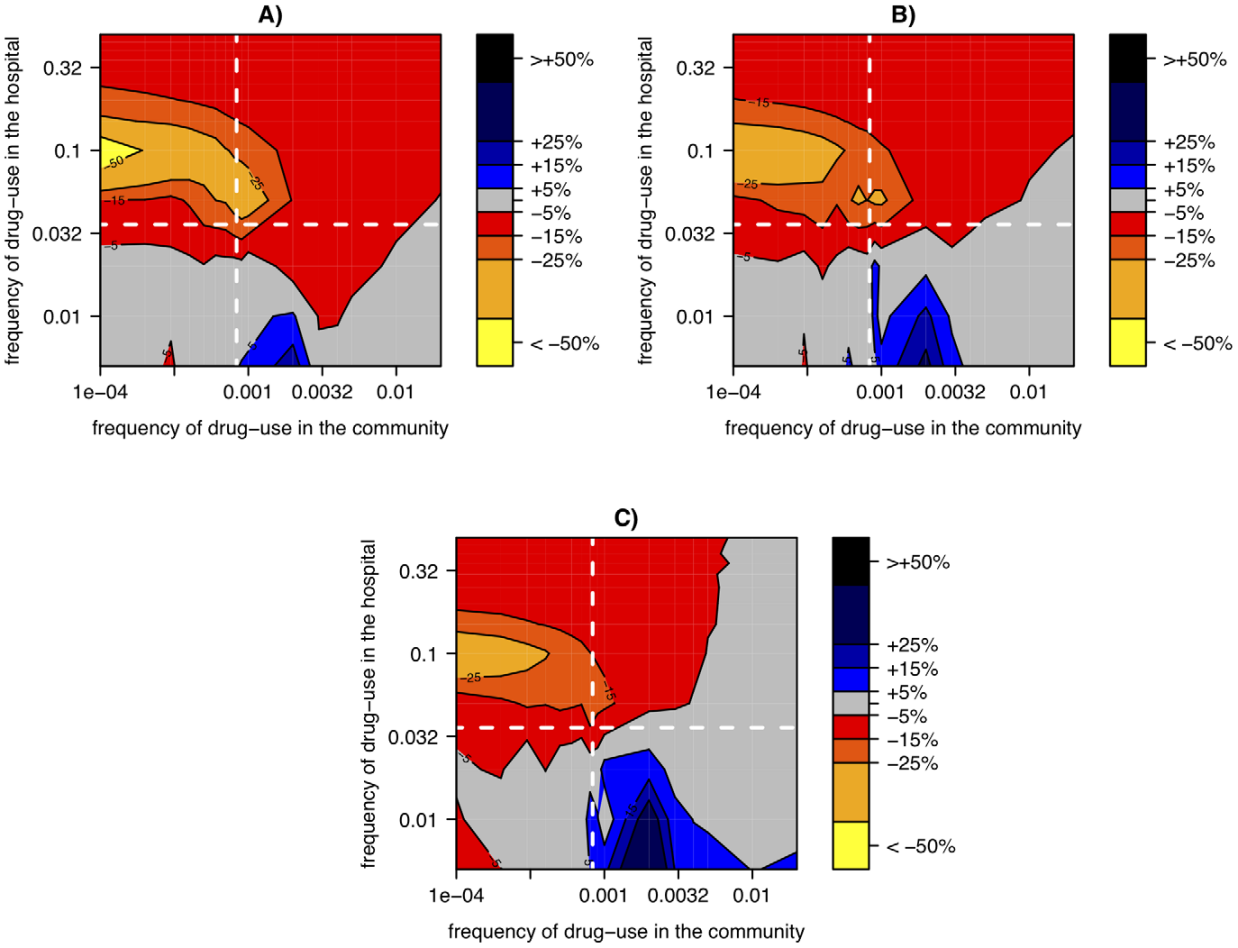
Relative change of resistance-prevalence in small vs. large hospitals linked to a subdivided community. The relative change in resistance-prevalence is measured as 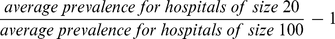 and is plotted as a function of the frequency of drug-use in the hospital and the community. Panels correspond to different migration rates between sub-communities: *m = 1%/year (A), m = 5%/year (B), m = 20%/year (C).* The cost of resistance was set to 0.2 (corresponding to Figure 6B). The resistance prevalence for each parameter combination has been computed as the average over 500 simulations.

If the community is subdivided as well, the beneficial effects of small hospital size are increased further (Figure 7). This is because, in most sub-communities, no resistant strains preexist when therapy is initiated. If in such a situation treatment frequencies are high enough, the entire bacterial population (i.e. all colonized individuals) in such a sub-community and the associated hospital might become extinct before the first resistant strain appears through mutation. In such a sub-community, all individuals will remain uncolonized until it acquires a resistant strain through migration from an other sub-community. Accordingly the additional effect conferred by substructures in the community strongly decreases as the migration rate between sub-communities increases (Figure 7b and c).

#### Effect of an environmental reservoir

**Figure 8:**
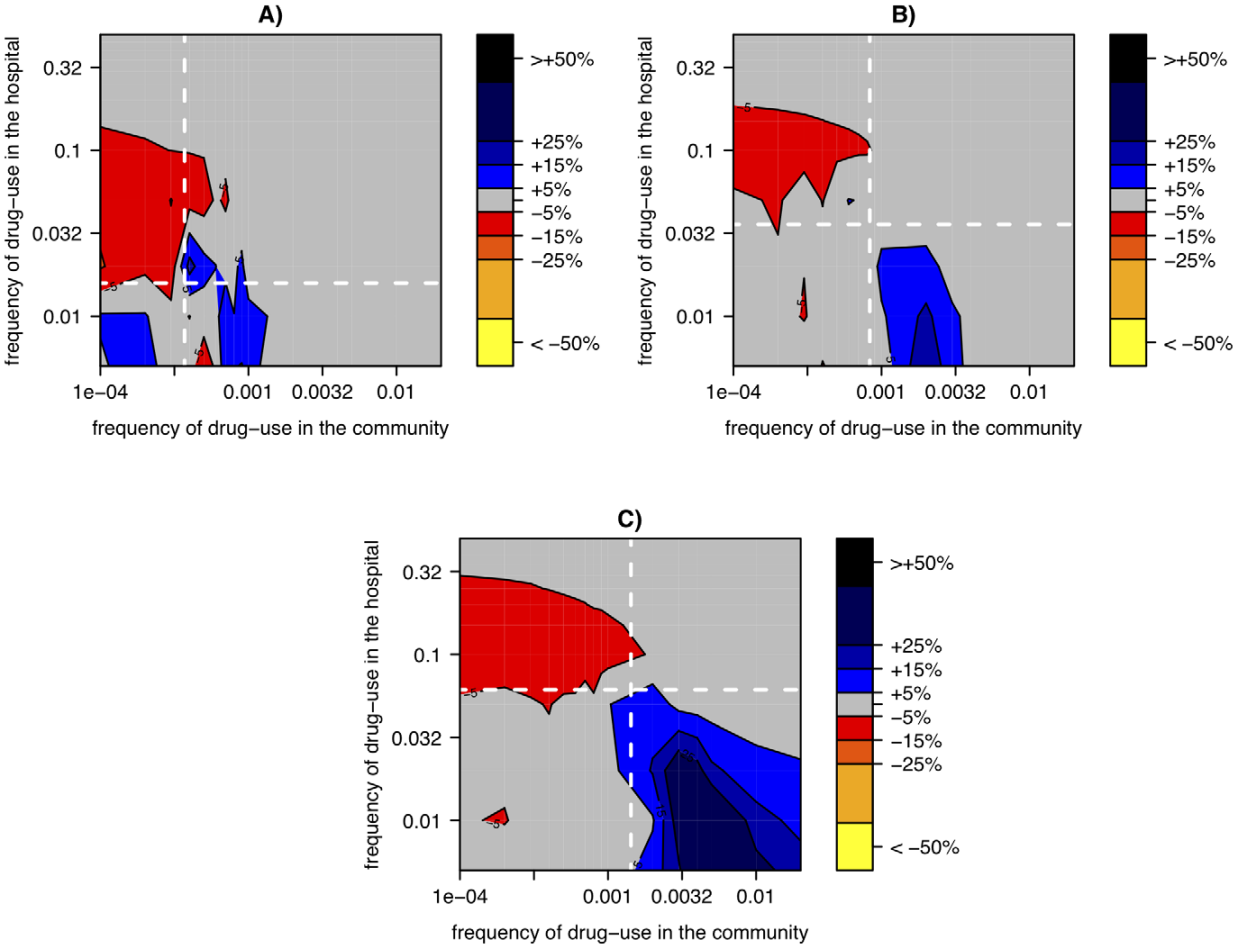
Relative change of resistance-prevalence in small vs. large hospitals with environmental transmission in the hospital. Same as Figure 6 but with environmental transmission in the hospital. Specifically, 50% of the force of infection in the hospital is mediated via the environmental reservoir. Panels correspond to different costs of drug resistance: *s = 0.1 (A), s = 0.2 (B), s = 0.3 (C).*

Nosocomial infections do not only spread by direct (patient-to-patient) transmission but also by indirect transmission via the environment (beds, doorknobs etc.). Hospitals exhibit a high turnover of patients and hence in the absence of influx or transmission, the number of infections decays rapidly and eventually the pathogen may become extinct. By contrast, the environment can potentially act as a reservoir [22] and thereby prevent extinctions. According to our above finding that small hospitals impede resistance evolution because of frequent extinctions, one would expect that the presence of environmental transmission should reduce this effect of hospital size. Indeed, we find that the addition of an environmental reservoir can considerably diminish the benefits of small hospital size. However, even in the presence of a reservoir, small hospitals can slightly reduce the burden of antibiotic resistance for many parameter settings (figure 8).

#### Effect of ward structure and intra-hospital transfer

**Figure 9:**
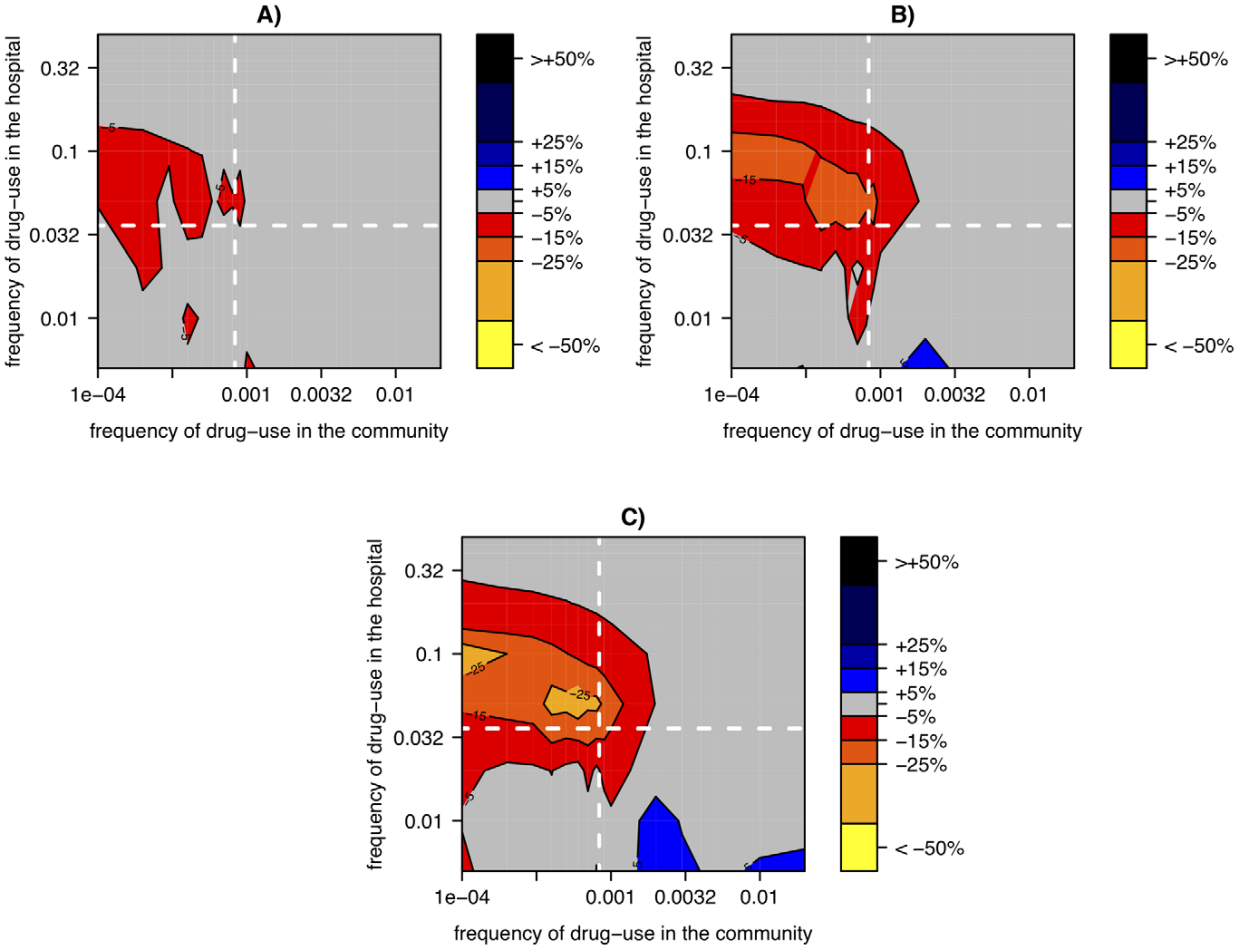
Relative change of resistance-prevalence in small vs. large subdivided hospitals. Same as Figure 6B, but assuming that the large hospital (size 100) is subdivided into 5 wards of 20 twenty with different transfer rate between wards (A: 0.01), (B: 0.05), (C: 0.1).

Up to this point we have approximated the patient population in a given hospital as panmictic; i.e. we have assumed that the transmission probability is the same for all pairs of patients. Hospitals, however, often exhibit strong population structure imposed by hospital wards. The opposite extreme of the panmictic hospital model considered so far is a hospital model consisting of completely subdivided hospital wards. This latter extreme is formally identical to a model in which every ward of a given size is replaced by hospital of the same size. The population structure of real hospitals most likely lies between these extremes: it is characterized by ward structure but also by frequent migration between wards. For instance Leverstein-VanHall et al. found, for patients infected with resistant strains, interward migration rates of 0.03–0.07 per patient-day[23]. We assessed this setting by comparing (as previously) the level of resistance in large hospitals (10 hospitals of 100 beds each) and in small hospitals (20 beds). Here, the large hospitals are subdivided into wards of 20 beds with inter-ward migration rates chosen between 0.01 and 0.1 per patient-day. With this model we find that on the one hand the disadvantage of large hospitals can be compensated in part by imposing a ward structure, but on the other hand we still find a substantial difference between large and small hospitals for realistic migration rates (see Figure 9).

## Discussion

It has been argued that concentrating highly specialized services in large hospitals will both improve patient outcomes as well as reduce health care costs [24], [25], [26], [27]. Yet, larger hospitals may have their own disadvantages by facilitating the spread of infectious diseases and resistance genes [10]. We used two surveillance datasets from the US [20] and Ireland [19] to illustrate how the incidence of infections and resistance depends on hospital size. In line with the results of Bhavnani et al. [10], we found that the rates of acquiring both resistant infections and infections in general strongly and significantly correlated with patient-days/year (hospital size). Furthermore, the increase of total infections over time and the ratio of resistant and drug-sensitive infections also significantly correlated with hospital size. Of course, different patient populations in small and large hospitals could also explain such a pattern. Antibiotic consumption and morbidity (and thereby susceptibility to infections) might be much higher in larger hospitals caring for more severely ill patients. However, this would not explain the steeper rise of infections in large hospitals. Additionally, the correlations between hospital size and incidence levels persisted even when taking the following potentially confounding factors into account: mortality, length of stay, antibiotic usage, usage of injectable or hospital-specific antibiotics, sampling frequency, or the usage of disinfectants. Both datasets analyzed have both problems and advantages. In the dataset from Ireland, for example, only S. aureus was considered, all positive blood cultures were recorded (even those which might have been acquired outside the given hospital) and there are no data on mortality. Conversely, the dataset from the US does not include the drug-sensitivity of the infections, drug usage or disinfection. However, these drawbacks do not overlap, and our results are coherent for both datasets. This makes it somewhat less likely that such confounders are the only reason for the correlation of resistance with hospital size. Furthermore, hospital size had a more significant impact than antibiotic usage on both relative prevalence and absolute incidence rates of methicillin-resistant S. aureus. Reducing antibiotic consumption is a common recommendation for curbing the spread of resistance. The analyzed data suggest that a reduction in hospital size might therefore be a similarly successful intervention.

We used a stochastic epidemiological model in order to describe both the spread of drug resistance in a hospital setting as well as the interaction between one or several hospitals and the community. Like all theoretical models, this is a very simplified description of complex interactions. For example, the epidemiological fitness costs of resistance are inherently difficult to determine. We used estimates of the epidemiological costs of resistance in tuberculosis [28] and of glycopeptide resistance in farm animals [29] as well as in vitro data [30] as guidelines, because no estimates on the transmissibility of resistant nosocomial infections exist, to our knowledge. Additionally, the model neglects co-infection, whereas in reality patients may harbor both resistant and sensitive strains, such that sufficiently long after discontinuation of antibiotic therapy the sensitive strains could dominate again. This effect can, however, be captured in our model by increasing the back-mutation rate (i.e. the rate with which a patient that is predominantly infected with the resistant strain becomes dominated by the sensitive strain in the absence of therapy). We find that increasing this rate substantially increases the magnitude of the effect of hospital size (results not shown). Intuitively, this is the case because with a higher reversion rate the level of resistance in the community (the “sink”) declines. Therefore it becomes less likely that the resistant strain is reintroduced from the community once it becomes extinct in a hospital. Thus, the assumption made here leads to rather conservative estimates for the effect of hospital size. In summary, the results from our theoretical model should be regarded as qualitative descriptions, not as quantitative predictions on how much resistance could be reduced if hospitals were smaller.

Theoretically, the impact of hospital size on the evolution of antibiotic resistance can be explained by the meta-population dynamics characterized by local extinctions and re-colonizations. According to this interpretation, the beneficial effect of small hospital size is the result of a simple evolutionary mechanism: Selection typically acts in different directions in the hospital and in the community: resistance is selected for in the hospital and selected against in the community. Thus any mechanism, which weakens the effectiveness of selection in the hospital relative to that in community, will lead to an increase in the level of resistance. According to Fisher's fundamental theorem, the effectiveness of selection acting on a trait (here trait  =  resistance) is proportional to the variance in that trait. Small hospital size decreases the variance in resistance through frequent extinctions of the resistance-conferring allele. In other words, if the resistance-conferring allele is extinct in a given hospital, selection (which would potentially favor this allele) is completely ineffective because the population consists only of the sensitive strain and selection can only favor one strain over the other if they coexist. In addition to the hospital-community setting described here, many more instances of population structure might cause similar meta-population effects that hinder the epidemic spread of antibiotic resistance mutations. Indeed, we made the same observations when considering inter-ward transfer. Further examples include: household structure, caring facilities, schools etc. Thus the effects described here are likely to extend beyond the hospital-community setting.

In this context it should be noted that for a real hospital of a given size, the magnitude of stochastic effects will typically be considerably stronger than what might be expected from a simple population-biological model like the one presented here. The reason for this is that several processes such as population structure, fluctuating population sizes, and variability in transmission rates (e.g. super-spreaders) strongly enhance stochasticity. Thus, a real hospital with a given number of patients will typically behave like an idealized hospital, which is much smaller. (This type of problem is well known in population genetics [31], and as a consequence populations are often characterized by a so-called effective population size rather than by their census population size). Nevertheless, all other things being equal, a small hospital will be subject to stronger stochastic effects than a large hospital and will hence suffer less from antibiotic resistance.

In contrast to the typical beneficial effect of small hospital size, we also found that under some circumstances small hospitals may increase the prevalence of antibiotic resistance. This effect occurs if antibiotic usage in the community reaches similar magnitude than in the hospital. Such a setting might be uncommon for nosocomial infections, which are mainly treated in the hospital. However, it might occur for other infections, which occur frequently in both the hospital and the community (e.g. *E. Coli* causes many opportunistic infections in the hospital and urinary tract infections in the community).

Overall, the results of this study suggest the general pattern that strong population subdivision in those compartments, where antibiotic usage is high (typically: the hospital), can substantially reduce the spread of antibiotic resistance. Furthermore, we find that this beneficial effect of small hospital-size is substantially reduced if a substantial fraction of infection events is not acquired directly from other patients (which exhibit a fast turnover in the hospital), but, indirectly, via a slowly decaying environmental compartment. This latter point indicates that reducing such environmental transmission might be especially important in small hospitals as this might not only reduce the force of infection but also the burden of antibiotic resistance by promoting the stochastic extinction of resistant pathogen strains.

## References

[1] Chambers HF, Deleo FR (2009). Waves of resistance: Staphylococcus aureus in the antibiotic era.. Nat Rev Microbiol.

[2] Neuhauser MM, Weinstein RA, Rydman R, Danziger LH, Karam G (2003). Antibiotic resistance among gram-negative bacilli in US intensive care units: implications for fluoroquinolone use.. JAMA.

[3] Abel zur Wiesch P, Kouyos R, Engelstädter J, Regoes R, Bonhoeffer S (2011). Population biological principles of drug-resistance evolution in infectious diseases.. Lancet Infect Dis.

[4] Smith DL, Levin SA, Laxminarayan R (2005). Strategic interactions in multi-institutional epidemics of antibiotic resistance.. Proc Natl Acad Sci U S A.

[5] Nicolle L (2001). Infection control programmes to contain antimicrobial resistance.. http://www.who.int/csr/resources/publications/drugresist/infection_control.pdf.

[6] Deleo FR, Otto M, Kreiswirth BN, Chambers HF (2010). Community-associated meticillin-resistant Staphylococcus aureus.. Lancet.

[7] Opatowski L, Mandel J, Varon E, Boelle PY, Temime L (2010). Antibiotic dose impact on resistance selection in the community: a mathematical model of beta-lactams and Streptococcus pneumoniae dynamics.. Antimicrob Agents Chemother.

[8] Goossens H, Ferech M, Vander Stichele R, Elseviers M (2005). Outpatient antibiotic use in Europe and association with resistance: a cross-national database study.. Lancet.

[9] Asensio A, Canton R, Vaque J, Rossello J, Calbo F (2006). Nosocomial and community-acquired meticillin-resistant Staphylococcus aureus infections in hospitalized patients (Spain, 1993-2003).. J Hosp Infect.

[10] Bhavnani SM, Hammel JP, Forrest A, Jones RN, Ambrose PG (2003). Relationships between patient- and institution-specific variables and decreased antimicrobial susceptibility of Gram-negative pathogens.. Clin Infect Dis.

[11] Chastre J (2008). Evolving problems with resistant pathogens.. Clin Microbiol Infect.

[12] Poirel L, Nordmann P (2006). Carbapenem resistance in Acinetobacter baumannii: mechanisms and epidemiology.. Clin Microbiol Infect.

[13] Zoutman DE, Ford BD (2005). The relationship between hospital infection surveillance and control activities and antibiotic-resistant pathogen rates.. Am J Infect Control.

[14] de With K, Meyer E, Steib-Bauert M, Schwab F, Daschner FD (2006). Antibiotic use in two cohorts of German intensive care units.. J Hosp Infect.

[15] Bergstrom CT, Lo M, Lipsitch M (2004). Ecological theory suggests that antimicrobial cycling will not reduce antimicrobial resistance in hospitals.. Proc Natl Acad Sci U S A.

[16] Bonhoeffer S, Lipsitch M, Levin BR (1997). Evaluating treatment protocols to prevent antibiotic resistance.. Proc Natl Acad Sci U S A.

[17] Gillespie DT (2001). Approximate accelerated stochastic simulation of chemically reacting systems.. J Chem Phys.

[18] Gillespie DT (1976). General Method for Numerically Simulating Stochastic Time Evolution of Coupled Chemical-Reactions.. J Comput Phys.

[19] Health Service Executive Ireland (2011). Healthcare-Associated Infection and Antimicrobial Resistance-Related Data from Acute Public Hospitals in Ireland, 2006-2007.. http://www.hse.ie/eng/services/newscentre/2008_Archive/May_2008/HSE_publishes_Health_Care_Associated_Infection_statistics.html.

[20] Pennsylvania Health Care Cost Containment Council (2011). Hospital-acquired Infections in Pennsylvania 2005, 2006 & 2007.. http://www.phc4.org/reports/hai/.

[21] Fisher RA (1930). The Genetical Theory of Natural Selection..

[22] Neely AN, Maley MP (2000). Survival of enterococci and staphylococci on hospital fabrics and plastic.. J Clin Microbiol.

[23] Leverstein-van Hall MA, Blok HE, Paauw A, Fluit AC, Troelstra A (2006). Extensive hospital-wide spread of a multidrug-resistant enterobacter cloacae clone, with late detection due to a variable antibiogram and frequent patient transfer.. J Clin Microbiol.

[24] Ahgren B (2008). Is it better to be big? The reconfiguration of 21st century hospitals: Responses to a hospital merger in Sweden.. Health Policy.

[25] Keeler EB, Rubenstein LV, Kahn KL, Draper D, Harrison ER (1992). Hospital Characteristics and Quality of Care.. JAMA.

[26] McCallion G, Glass JC, Jackson R, Kerr CA, McKillop DG (2000). Investigating productivity change and hospital size: a nonparametric frontier approach.. Appl Econ.

[27] Carr BG, Goyal M, Band RA, Gaieski DF, Abella BS (2009). A national analysis of the relationship between hospital factors and post-cardiac arrest mortality.. Intensive Care Med.

[28] Luciani F, Sisson SA, Jiang H, Francis AR, Tanaka MM (2009). The epidemiological fitness cost of drug resistance in Mycobacterium tuberculosis.. Proc Natl Acad Sci U S A.

[29] Johnsen PJ, Townsend JP, Bohn T, Simonsen GS, Sundsfjord A (2011). Retrospective evidence for a biological cost of vancomycin resistance determinants in the absence of glycopeptide selective pressures.. J Antimicrob Chemother.

[30] Trindade S, Sousa A, Xavier KB, Dionisio F, Ferreira MG (2009). Positive epistasis drives the acquisition of multidrug resistance.. PLoS Genet.

[31] Hartl DL, Clark AG (2007). Principles of Population Genetics..

[32] Lucet JC, Paoletti X, Demontpion C, Degrave M, Vanjak D (2009). Carriage of methicillin-resistant Staphylococcus aureus in home care settings: prevalence, duration, and transmission to household members.. Arch Intern Med.

[33] van Loon HJ, Vriens MR, Fluit AC, Troelstra A, van der Werken C (2005). Antibiotic rotation and development of gram-negative antibiotic resistance.. Am J Respir Crit Care Med.

[34] Cars O, Molstad S, Melander A (2001). Variation in antibiotic use in the European Union.. Lancet.

